# Mechanical Analysis of Romosozumab's Effects on Bone Strength in a Rat Posterolateral Lumbar Fusion Model

**DOI:** 10.7759/cureus.79802

**Published:** 2025-02-27

**Authors:** Tomohito Mukaihata, Kazuhide Inage, Yasuhiro Shiga, Geundong Kim, Ikuko Tajiri, Noriyasu Toshi, Miyako Suzuki-Narita, Masahiro Inoue, Seiji Ohtori, Sumihisa Orita

**Affiliations:** 1 Spine Center, Katori Omigawa Medical Center, Katori, JPN; 2 Department of Orthopaedic Surgery, Graduate School of Medicine, Chiba University, Chiba, JPN; 3 Department of Orthopaedic Surgery, Minamigyotoku Orthopedic and Internal Medicine Clinic, Tokyo, JPN; 4 Department of Orthopaedic Surgery, Chiba Prefectural Sawara Hospital, Katori, JPN; 5 Department of Bioenvironmental Medicine, Graduate School of Medicine, Chiba University, Chiba, JPN; 6 Center for Frontier Medical Engineering, Chiba University, Chiba, JPN

**Keywords:** bone healing advances, bone strength, osteoporosis, romosozumab, spinal fusion surgery

## Abstract

Purpose: This study aimed to evaluate the effects of romosozumab, a humanized monoclonal antibody, on bone healing and mechanical strength in a rat posterolateral lumbar fusion (PLF) model. The primary objective was to determine its potential in promoting bone union and enhancing the structural integrity of graft sites, addressing challenges such as pseudarthrosis and hardware failure in spinal surgeries. These complications are particularly common in osteoporotic patients, where compromised bone quality and reduced healing capacity significantly increase the risk of surgical failure. With an aging global population, osteoporosis-related complications in spinal surgery are expected to rise, necessitating novel interventions to improve outcomes.

Materials and methods: Twenty male Sprague-Dawley rats were randomized into two groups: romosozumab-treated (R) and control (C). All animals underwent bilateral PLF surgery involving the placement of autogenous bone grafts harvested from the spinous process combined with a demineralized bone matrix between the transverse processes of the lumbar vertebrae. Subcutaneous injections of romosozumab (105 mg/1.17 mL) or saline were administered twice weekly for 10 weeks. Bone healing was assessed through micro-computed tomography (CT) imaging at baseline and 10 weeks post-surgery. Key metrics included the bone fusion rate, fused bone volume, and bone mineral density (BMD). Additionally, the mechanical strength of the fusion mass was evaluated using a three-point bending test to determine the force required to induce rupture.

Results: The R group exhibited significant improvements across all evaluated parameters compared to the C group. Fused bone volume in the R group was significantly greater at 10 weeks (826.7 ± 27.5 mm³) compared to the C group (652.6 ± 30.7 mm³, p < 0.05), reflecting a higher bone volume growth rate (158.1 ± 12.9% vs. 106.8 ± 10.4%, p < 0.05). BMD at the distal femoral diaphysis was also markedly increased in the R group (830.2 ± 11.1 mgHA/cm³) compared to the C group (725.5 ± 12.1 mgHA/cm³, p < 0.05). Mechanical testing revealed superior compressive strength in the R group, with a rupture force of 312.5 ± 43.2 N versus 209.3 ± 35.4 N in the C group (p < 0.05). These results demonstrate romosozumab's capacity to promote robust bone formation and significantly enhance the mechanical integrity of the fusion mass.

Conclusion: Romosozumab treatment significantly improved bone healing, mineral density, and mechanical strength in a rat PLF model, suggesting its potential as a therapeutic option for enhancing spinal surgery outcomes. By promoting rapid bone formation and increasing bone strength, romosozumab addresses critical challenges such as pseudarthrosis and pedicle screw loosening, which frequently compromise surgical success, especially in osteoporotic patients. These findings underscore the therapeutic promise of romosozumab not only in spinal surgery but also as a broader intervention for bone repair and healing. Further research is needed to explore its dose-response relationship, long-term safety, and efficacy in osteoporotic models. Moreover, the use of biochemical markers and microstructural analyses will help elucidate the underlying mechanisms of its action. With its demonstrated ability to enhance both structural and functional bone properties, romosozumab offers a promising avenue for advancing spinal surgery and improving patient outcomes.

## Introduction

In recent years, many spinal fusion surgeries have been performed on patients with osteoporosis against the backdrop of an aging society. In such cases, postoperative vertebral fractures and pedicle screw (PS) loosening are often observed, which are serious postoperative complications. A cadaver study indicated that PS loosening is more likely to occur in patients with osteoporosis due to low pull-out strength [1]. Furthermore, it is expected that the number of spine surgeries for patients with severe osteoporosis will increase in the future. Therefore, there is an urgent need to prevent postoperative complications and achieve good postoperative results by promoting rapid bone healing and strengthening following surgery.

Various osteoporosis drugs (bisphosphonates, human parathyroid hormone, etc.) that act on bone remodeling and improve bone strength are already in use. Bone remodeling comprises three stages: osteoclast initiation of bone resorption (bone resorption stage), bone resorption completion and transition to the bone formation by osteoblasts (transition stage), and new bone formation (bone formation stage) [2]. This process is consistent with bone healing after spinal surgery, and the effects of various osteoporosis drugs on bone healing have been reported in recent years [3,4].

Romosozumab has attracted particular attention. It is a humanized immunoglobulin monoclonal antibody that binds to and inhibits sclerostin, an extracellular inhibitor of the classical Wnt signaling pathway secreted by osteocytes that inhibits bone formation. Romosozumab specifically binds to sclerostin and inhibits the suppression of Wnt signaling in osteoblast lineage cells by preventing sclerostin from binding to LRP5 and LRP6 [5,6]. In the context of bone healing, this mechanism is particularly relevant as the Wnt/β-catenin pathway plays a crucial role in multiple stages of fracture repair and bone formation. During the early stages of bone healing, Wnt signaling promotes mesenchymal cell differentiation toward the osteoblast lineage rather than the chondrocyte lineage. In later stages, it enhances osteoblast proliferation, differentiation, and function while inhibiting osteoclastogenesis. By blocking sclerostin, romosozumab effectively removes the "brake" on this pathway, potentially accelerating and enhancing the bone formation phase of healing. This dual anabolic-antiresorptive effect distinguishes romosozumab from other osteoporosis therapies and makes it particularly interesting for applications in bone healing and fusion procedures. The resulting activation of classical Wnt signaling increases bone formation, decreases bone resorption, and increases bone mass and strength in cortical and trabecular bone. Pre-marketing clinical trial data have also demonstrated that romosozumab stimulates bone formation and inhibits bone resorption. For example, in the FRAME study, an average decrease rate of approximately 35% in bone resorption (CTX) and formation markers (P1NP) one month after romosozumab treatment (statistically significant improvement compared with pre-treatment) reportedly increased by an average of approximately 95% (statistically significant improvement compared with that before treatment) [7].

Based on the above evidence, romosozumab is naturally expected to promote bone healing and increase bone strength. However, since it has only been introduced to Japan recently, there have been few reports on its therapeutic effects in actual clinical practice. Therefore, the objectives of this study were as follows: (1) to evaluate the effects of romosozumab administration on bone healing and mechanical strength in a rat posterolateral lumbar fusion (PLF) model and (2) to assess changes in bone volume, bone mineral density, and compressive strength as measured by three-point bending tests following romosozumab treatment compared to control.

We hypothesized that romosozumab administration would enhance bone formation, increase bone mineral density, and improve the mechanical integrity of the fusion mass in the experimental model.

This article was previously posted as a preprint to Research Square on September 2, 2022.

## Materials and methods

Experimental animals

A total of eight-week-old male Sprague-Dawley rats (n = 20; 200-250 g; Japan SLC Inc., Shizuoka, Japan) were used in this study. We conducted this study in compliance with the principles of the Declaration of Helsinki. This study was approved by the Ethics Approval Committee of Chiba University School of Medicine (issued protocol number A6-039).

Posterolateral lumbar fusion surgery

**Figure 1:**
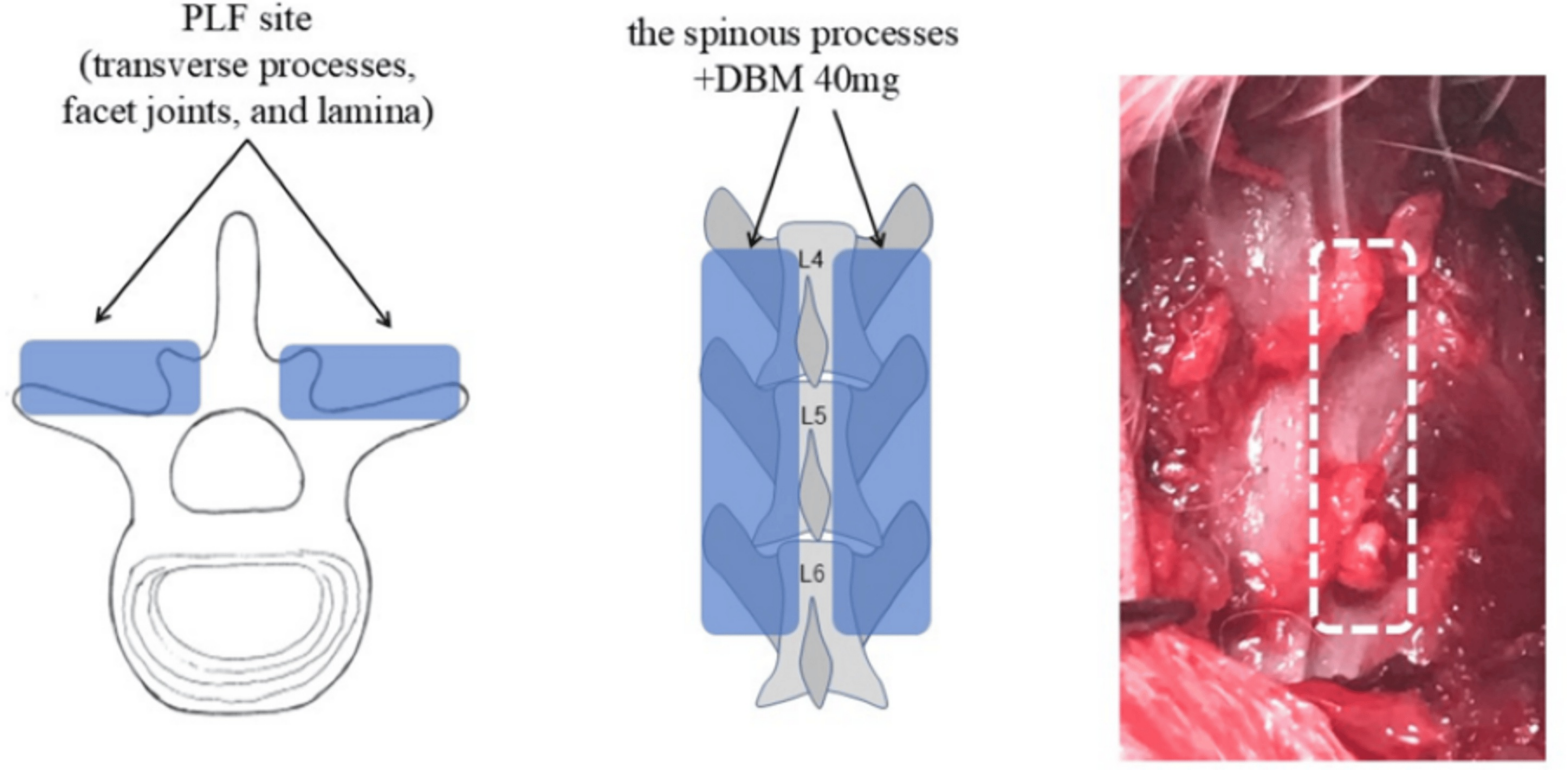
The fourth to sixth PLF surgery. A shows the bone graft site in an axial section; B shows the bone graft site in a coronal section; and C shows the bone graft site in a gross image. PLF, posterolateral lumbar fusion. All images are created by the authors.

All rats were injected intraperitoneally with a mixture of three anesthetic agents, namely, Domitor 0.15 mL/kg (Nippon Zenyaku Kogyo Co., Ltd., Japan), Dormicum 2 mg/kg (Astellas Pharma Inc., Japan), and Vetorphale 2.5 mg/kg (Meiji Seika, Ltd., Japan), diluted in saline 1.45 mL/kg (Otsuka Pharma Inc., Japan). Subsequently, ampicillin sodium (20,000 U/kg; Meiji Seika Co., Ltd., Tokyo, Japan) was administered subcutaneously before surgery [8]. A skin incision was made in the mid-dorsal region, and the paraspinal fascia was incised bilaterally to expose the fourth, fifth, and sixth lumbar vertebral arches and transverse processes and the fourth/fifth and fifth/sixth lumbar intervertebral joints. Forty milligrams of graft bone were taken from the spinous process of the 10th thoracic vertebra to the second lumbar vertebra, and 40 mg of demineralized bone matrix (Medtronic, Memphis, TN) was placed between the facet joints and transverse processes on both sides [9-11], as an autogenous and artificial bone graft (Figure 1).

The fascia and skin were sutured using 4-0 absorbable sutures. Following surgery, rats were housed individually in polycarbonate cages (42 × 26 × 18 cm), with soft bedding material to minimize discomfort. The animal facility was maintained at a controlled temperature (22 ± 2 °C) and humidity (55 ± 10%), with a 12-hour light/dark cycle. Animals had ad libitum access to standard laboratory rodent diet and water. For postoperative care, we administered buprenorphine (0.05 mg/kg, subcutaneously) for pain management every 12 hours for the first 48 hours post surgery. Surgical wounds were inspected daily for the first week to monitor for signs of infection or complications. Animals were weighed weekly to monitor general health throughout the study period. Postoperatively, all rats were kept in cages and could eat and drink freely [8].

Experimental groups

To eliminate differences in body size, the animals were divided into two groups based on body weight: the romosozumab (R group) and control groups (C group) (with 10 animals each). To prevent bias in outcome assessment, investigators performing the evaluations (CT image analysis, bone densitometry, and mechanical strength tests) were blinded to the group allocation throughout the study. The allocation sequence was secured by a researcher who was not involved in the outcome assessments.

The R group was treated with romosozumab (anti-sclerostin antibody: 105 mg/1.17 mL, calcium acetate hydrate: 2.41 mg/1.17 mL (13 mM), acetic anhydride: 2.04 mg/1.17 mL (17 mM), sucrose 70 mg/1.17 mL (6%), polysorbate20: 0.070 mg/1.17 mL (0.006%), pH 5.2; Amgen, Thousand Oaks, CA, USA). The C group was administered saline (25 mg/kg; Otsuka Pharmaceutical Co., Ltd.). The injections were administered subcutaneously at the dorsal neck region, with a volume of 1.17 mL per injection, using a 25-gauge needle. The injection solutions were stored at 2-8 °C and brought to room temperature approximately 30 minutes before administration. Care was taken to maintain sterile conditions throughout the injection procedure. The injections were administered subcutaneously twice a week (Tuesdays and Fridays, in the morning) for 10 weeks [11,12].

The romosozumab dosage (105 mg/1.17 mL) was selected based on previous experimental studies in rat models [11] that demonstrated effective sclerostin inhibition at this concentration. While this dose is higher than clinical doses when converted to human equivalence, it has shown consistent results in preclinical research examining bone formation effects.

The first dose of romosozumab was administered immediately after the completion of PLF surgery, with subsequent doses given twice weekly for 10 weeks.

Evaluation tests

Micro-computed Tomography Examination

**Figure 2:**
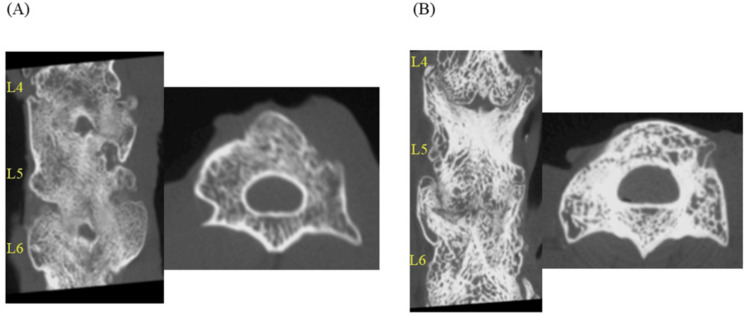
Representative CT (coronal and axial sections) at week 10 post surgery. A: C group; B: R group. CT, computed tomography

The bone graft site was imaged immediately after surgery using a micro-computed tomography (CT) system (in vivo micro-CT system, R_mCT2; Rigaku Corporation, Tokyo, Japan; 30-mm field of view, and 26-s exposure time). After euthanasia by an overdose of a three-component mixed anesthetic at 10 weeks postoperatively, the lumbar spine was removed, and the images were taken again (Figure 2).

The acquired image data were evaluated by three spine surgeons who were unrelated to this study to assess the rate of bony fusion between the facet joints and transverse processes in the two groups. In each of the intervertebral levels, the left and right intervertebral joints are evaluated as one site each. Bone fusion was determined based on the following criteria: (1) the presence of a continuous bone bridge connecting the transverse processes, (2) the absence of radiolucent gaps within the fusion mass, and (3) the density of the fusion mass similar to or greater than the adjacent bone. At least two of these three criteria had to be satisfied for a site to be considered fused. Each site was independently evaluated by three surgeons, and a site was considered fused when at least two surgeons agreed on the fusion status. Bone fusion was determined if there was a finding of bony continuity. In addition, to qualitatively evaluate bone fusion, the volume of fused bone in both groups was measured and compared using Analyze 12 software (AnalyzeDirect, Overland Park, KS) and Ziostation2 software (Ziosoft Inc., Tokyo, Japan).

Bone Densitometry

After euthanasia, the right femur neck was removed and imaged using the aforementioned CT system. Bone mineral density (BMD (mg HA/cm^3^)) was measured from the obtained image data using dedicated software (Bone Analysis software; Rigaku Co., Ltd. Austin, TX) [1,8,9].

Mechanical Strength Examination (Three-Point Bending Test)

**Figure 3:**
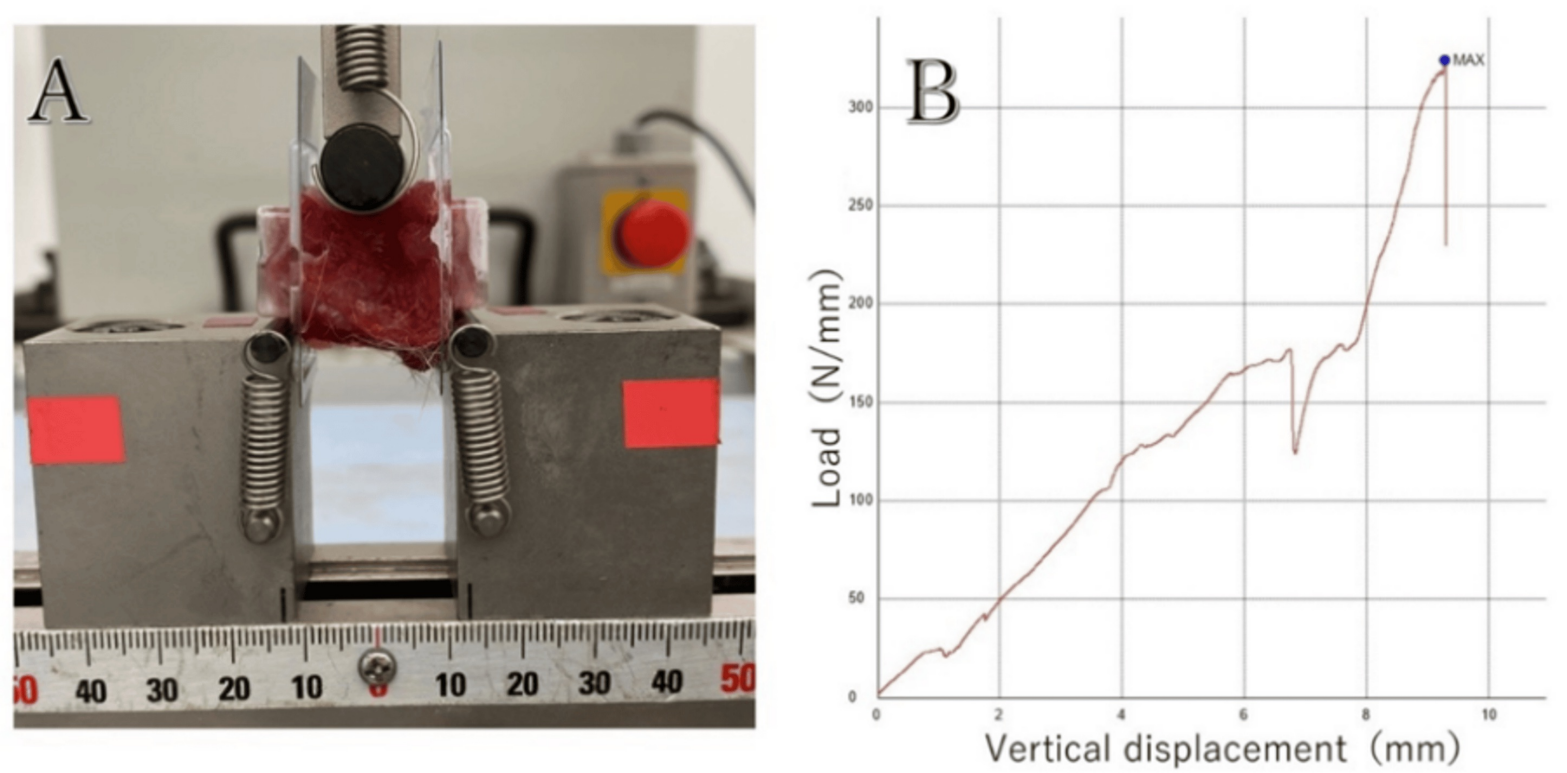
Mechanical strength evaluation: three-point bending test. A: Three-point bending of the harvested lumbar spine (L4–L6). B: Representative plotting for the initial peak pressure measurement.

L4-L6 lumbar spine specimens (3.5 cm long) were collected from rats, fixed on both sides with plastic holders (Cryomold #2; Sakura Finetek, Tokyo, Japan), and set on a three-point bending test apparatus (Shimadzu, Tokyo, Japan). The apparatus consisted of two lower supports set 25 mm apart and one upper loading point positioned centrally between the supports. The specimens were positioned so that the ventral side of the spine rested on the lower supports and the dorsal side (with the fusion mass) faced the upper loading point. The orientation was verified before each test to ensure consistency across all specimens. The compression strength of the dorsal side of the spine with PLF was evaluated by fixing the ventral side at two points and the dorsal side at one point and applying force. The upper loading point was connected to a load cell with a maximum capacity of 500 N and an accuracy of ±0.1 N. The displacement rate was set at 0.1 mm/s with data sampling at 50 Hz. Load-displacement curves were recorded for each test, and analysis software (Trapezium X; Shimadzu, Tokyo, Japan) was used to calculate mechanical parameters. To avoid changes in stiffness over time, the specimens were brought to room temperature on the day of euthanasia, and pressure was continuously applied to the specimens while monitoring them, in real time. No rotation or slippage of the specimens was observed during the experiment. Bones were preloaded with 10 N/mm^2^ of force at a rate of 0.1 mm/s and allowed to acclimate for 10 s [13]. We defined mechanical strength as newtons at the point of rupture (Figure 3).

After mechanical strength measurements, CT imaging was performed to confirm the fracture site.

Statistical analyses

Data are presented as the mean ± standard deviation. Data comparisons for each group were statistically analyzed using an unpaired t-test, and statistical significance was set at p < 0.05.

## Results

In this study, no adverse effects were observed. Table 1 shows the results of the computed tomography examination and the three-point bending test (Table 1).

**Table 1:** Results of computed tomography examination and the 3-point bending test, analyzed using an unpaired t-test.

	Romosozumab	Control	p-value
Number of rats	10	10	-
Bone union rate (%)	100±0	92±5.6	0.539
Average volume of PLF			
0th week (mm^3^)	320.3±11.4	315.6±18.7	0.701
10th week (mm^3^)	826.7±27.5	652.6±30.7	< 0.05
growth rate (%)	158.1±12.9	106.8±10.4	< 0.05
Bone density (mgHA/cm^3^)	830.2±11.1	725.5±12.1	< 0.05
Mechanical strength (N)	312.5±43.2	209.3±35.4	< 0.05

CT examination

The mean bone fusion rate between the facet joints and transverse processes tended to be higher in the R group (100 ± 0%) than that in the C group (92 ± 5.6%), though the difference was not statistically significant. The volume of fused bone did not significantly differ between the two groups immediately after surgery (315.6 ± 18.7 mm^3^ and 320.3 ± 11.4 mm^3^ for the C and R groups, respectively). However, at 10 weeks after surgery, the R group has a significantly higher fused bone volume (826.7 ± 27.5 mm3) than the C group (652.6 ±30.7 mm^3^) (p < 0.05).

The increase in bone fusion volume was also significantly greater in the R group (158.1 ± 12.9%) than that in the C group (106.8 ± 10.4%) (p < 0.05).

Bone densitometry

The mean bone mineral density of the distal femoral diaphysis was significantly greater in the R group (830.2 ± 11.1 mgHA/cm^3^) than that in the C group (725.5 ± 12.1 mgHA/cm^3^) (p < 0.05).

Mechanical strength examination

**Figure 4:**
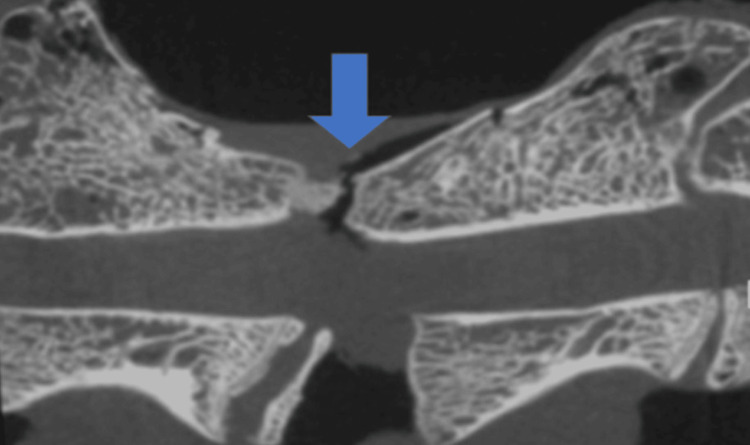
CT after a three-point bending test. The blue arrowhead indicates the fracture site in the control group. CT, computed tomography

Strength measurements revealed that the R group (312.5 ± 43.2 N/mm^2^) had a significantly higher mean value of force at rupture relative to that of the C group (209.3 ± 35.4 N/mm^2^) (p < 0.05). On CT after fracture, the fracture site was found on the endplate of the vertebral cartilage in all cases (Figure 4).

## Discussion

Bone fusion rate

In this study, the mean bone fusion rate was 92% and 100% in the C and R groups, respectively. Although there was no significant difference, the R group tended to have a higher bone healing rate than the C group. In the micro CT analysis of a rat PLF model and femur fracture, it has been reported that the bone healing rate at the fracture site was superior in romosozumab-treated groups than that in control groups due to romosozumab’s considerable bone formation effect in the rat bone fracture model [14,15].

In the present study, a higher bone healing rate was also achieved with romosozumab treatment.

Volume of the bone fusion area

The mean volume of fused bone was significantly higher in the R group at 10 weeks postoperatively, although there were no differences between the two groups preoperatively.

In a different rat model from that used in the present study, Kim et al. found that the fracture volume in a romosozumab-treated group was significantly larger than that in the control group by 13.5% in a micro-CT analysis of a rat spinal PLF model eight weeks postoperatively [15]. Furthermore, van Bezooijen et al. reported that the fracture volume in a romosozumab-treated group was significantly larger than that in the control group eight weeks postoperatively in a micro-CT analysis of a rat femur extension model. In addition, the same authors reported a significantly larger volume of regenerated bone in the romosozumab-treated group than that in the control group by 26-38% [16].

This suggests that romosozumab treatment promotes bone augmentation at the site of bone grafting, which may be attributed to the modeling effect of romosozumab. In addition, romosozumab specifically binds to sclerostin and inhibits classical Wnt signaling in osteoblast lineage cells by preventing sclerostin from binding to LRP5 and LRP6. The resulting activation of classical Wnt signaling reportedly increases bone formation, decreases bone resorption, and increases bone mass in cortical and trabecular bone (modeling) [12,17].

Therefore, it is feasible that similar modeling can be promoted in bone grafts as in the present study, and this effect could have increased the mean volume of the bone fusion zone in the R group compared with that in the C group.

Bone density

In the present study, the bone mineral density of the proximal femur increased significantly in the R group relative to that in the C group. Several studies have reported a romosozumab-induced increase in bone mineral density [5,15], consistent with the present study. Therefore, our study reinforces the effect of romosozumab administration on bone mineral density.

Mechanical strength examination (three-point bending test)

Mechanical testing using the three-point bending method revealed that, when subjected to a compressive force, bone strength at the site of PLF was predominantly higher in the R group than that in the C group. Similarly, based on finite element analysis using CT images, Brown et al. reported that one year of romosozumab treatment, followed by alendronate administration, increased bone strength in postmenopausal women at high risk of osteoporosis compared with those treated with alendronate only [18]. Furthermore, in a subanalysis of the FRAME study, bone volume and microstructure at 12 months, as assessed by micro-CT, were significantly increased with romosozumab administration [19]. Bone mineral density and trabecular microstructure are the strongest predictors of bone strength [20]. Therefore, we believe that the results of this study demonstrate that romosozumab treatment increases bone strength not only in the treatment of osteoporosis but also in the bone graft area following spine surgery through vigorous bone remodeling and good bone beam formation.

The consistent occurrence of fractures at the vertebral endplate in both groups warrants further discussion. This pattern might be explained by several factors: First, the endplate represents a natural transition zone between different tissue types (cortical and cancellous bone), making it potentially more susceptible to mechanical stress. Second, while romosozumab enhanced overall bone strength, the differential response of various bone regions to the treatment might create local variations in mechanical properties. The higher bone formation rate in the fusion mass compared to the endplate region could potentially lead to stress concentration at this interface. This observation suggests the importance of considering regional variations in bone remodeling when evaluating therapeutic effects on mechanical properties.

Strength measurements revealed that the R group (312.5 ± 43.2 N/mm^2^) had a significantly higher mean value of force at rupture relative to that of the C group (209.3 ± 35.4 N/mm^2^) (p < 0.05). On CT after fracture, the fracture site was found on the endplate of the vertebral cartilage in all cases (Figure 4). This consistent fracture pattern at the vertebral endplate across all specimens warrants further discussion. The fact that failure occurred at the endplate rather than through the fusion mass in both groups suggests that the fused bone developed sufficient strength to transfer the load to the adjacent vertebral structures. This pattern indicates that romosozumab not only enhanced the fusion mass strength but did so to the extent that the vertebral endplate became the mechanical weak point of the motion segment. From a clinical perspective, this finding is significant as it suggests that romosozumab-enhanced fusion may provide sufficient stability to shift the failure point away from the fusion site itself. Additionally, this observation aligns with previous studies showing that romosozumab increases bone strength throughout the skeleton, not just at the specific treatment site [16,18]. Future studies could investigate whether romosozumab also strengthens the vertebral body and endplate structures, which would be particularly valuable in osteoporotic patients where vertebral fractures commonly occur at these locations.

Adverse effect considerations

While our study demonstrated the positive effects of romosozumab on bone healing and strength, it is important to acknowledge the potential adverse effects associated with this treatment. In clinical trials, romosozumab has been associated with an increased risk of cardiovascular events, particularly myocardial infarction and stroke [2]. Although we did not observe any obvious adverse effects in our experimental animals during the study period, we did not specifically monitor for cardiovascular complications. Other reported adverse effects in humans include injection site reactions, headache, arthralgia, and rare cases of osteonecrosis of the jaw and atypical femoral fractures, though these occur less frequently than with long-term bisphosphonate use [21]. The safety profile of romosozumab in the context of perioperative administration for enhancement of spinal fusion requires further investigation in larger animal models and eventually in clinical trials. The risk-benefit ratio should be carefully considered, particularly in patients with pre-existing cardiovascular risk factors. Additionally, while the dosage used in our study was based on previous experimental protocols, dose-optimization studies would be valuable for determining the minimal effective dose that maximizes bone healing while minimizing potential adverse effects.

Comparison with established treatments and long-term considerations

Considering our findings in the context of established treatments for enhancing spinal fusion, several comparisons are noteworthy. Bisphosphonates, which inhibit osteoclastic bone resorption, have shown variable effects on spinal fusion in animal models and clinical studies. While they preserve bone mass, their anti-remodeling effects may potentially impair the early phases of bone healing [22]. In contrast, anabolic agents such as teriparatide (PTH 1-34) have demonstrated positive effects on spinal fusion rates comparable to our observations with romosozumab. Ohtori et al. reported that teriparatide administration enhanced posterolateral lumbar fusion in osteoporotic women with a fusion rate of 84% compared to 74% in the control group [4]. Our results suggest that romosozumab may produce similar or potentially greater improvements in fusion outcomes, though direct comparative studies are needed to confirm this hypothesis.

The long-term sustainability of romosozumab's effects on bone fusion is an important consideration not addressed in our 10-week study. Clinical studies of romosozumab in osteoporosis have shown that its bone-forming effects diminish after 12 months, suggesting a potential "anabolic window" [23]. Upon discontinuation, there is typically a gradual decline in bone mineral density unless followed by an anti-resorptive agent. In the context of spinal fusion, it remains unclear whether short-term administration during the critical early healing phase would provide sustained benefits. Future studies with longer follow-up periods (6-12 months) would be valuable to determine if the enhanced fusion mass maintains its integrity over time.

Regarding mechanistic insights, while our study demonstrates the effects of romosozumab on bone healing outcomes, additional analyses of bone microarchitecture and molecular markers would provide a deeper understanding of the underlying biology. The Wnt/β-catenin pathway affected by romosozumab influences not only bone mass but also bone quality through effects on mineralization, collagen organization, and osteocyte function [24]. Future studies incorporating histomorphometry, gene expression analysis of Wnt target genes, and assessment of serum markers of bone formation (P1NP) and resorption (CTX) would elucidate the specific cellular and molecular mechanisms by which romosozumab enhances spinal fusion.

Limitations

There are some limitations to this study. First, the dose of romosozumab is considerably higher than that used in clinical practice (approximately 19-fold higher in human equivalence) [25]. This significant dose difference was based on previously established experimental protocols in rodent models that demonstrated measurable effects on bone metabolism. However, this high dose raises important questions about translation to human applications. The dose-response relationship for romosozumab in the context of bone healing may differ from that for the treatment of osteoporosis, and supraphysiological doses might produce effects that would not be observed with clinically approved dosages. Therefore, caution should be exercised when extrapolating our results to human clinical scenarios. Since this study was based on the dosage from a basic experiment in a previous similar study, additional experiments should be conducted in the future to determine if the outcomes are dose-dependent [1].

Ideally, future studies should include multiple dosage groups to establish a dose-response curve that could better inform potential therapeutic applications. Second, we did not use an osteoporosis model in the present study. Since we referred to the aforementioned basic experiment, we plan to conduct additional experiments using ovariectomized (OVX) rats in the future. Third, the effects of romosozumab on bone mineral density were evaluated only at the last observation. This represents an important limitation as we were unable to provide a comparison of pre- and post-treatment bone density data, which would have allowed a more direct assessment of the treatment effect on bone density over time. A longitudinal analysis of bone density changes would provide a more comprehensive understanding of romosozumab's temporal effects on bone metabolism. In future studies, the use of in vivo micro-CT scanning at multiple time points (e.g., baseline, two weeks, five weeks, and 10 weeks) would enable such serial measurements without requiring euthanasia at each time point. A shortcoming of our bone densitometry system is that it is limited by the size of the subject and requires the euthanasia of the subject. Therefore, we are considering additional experiments to examine the effects of romosozumab before and after administration using bone metabolism markers. Fourth, pathological evaluation was not possible due to tissue destruction during three-point bending, and the percentage of bone trabeculae could not be evaluated. We are considering conducting an additional experiment to evaluate the pathology without three-point bending. Fifth, only the compressive strength of the area where PLF was performed was evaluated in the mechanical tests. Since loads are applied variously in actual clinical practice, we are considering evaluating the extensional strength of the fusion site by applying three-point bending from the opposite direction.

While our study focuses on the short-term effects of romosozumab on bone healing, it is important to consider the potential long-term implications. Romosozumab's mechanism of action suggests that sustained bone formation and reduced bone resorption could lead to improved bone quality and reduced fracture risk over time. However, further research is needed to evaluate the long-term safety and efficacy of romosozumab in spinal fusion.

## Conclusions

In this study, we investigated the effect of romosozumab administration on bone healing and strength in a rat PLF model. Results indicated that fused bone volume was significantly higher in the R group at the last observation of the fused bone. The rate of fused bone volume increase was also significantly higher in the R group. The strength of the PLF area when subjected to compression from the three-point bending test was also significantly higher in the R group than that in the C group. Therefore, these results suggest that romosozumab treatment promotes bone healing and increases bone strength. This study may contain important findings in establishing new methods of preventing spinal surgery complications associated with bone fusion failure. Romosozumab treatment significantly improved bone healing, mineral density, and mechanical strength in a rat PLF model, suggesting its potential as a therapeutic option for enhancing spinal surgery outcomes. By promoting rapid bone formation and increasing bone strength, romosozumab addresses critical challenges such as pseudarthrosis and pedicle screw loosening, which frequently compromise surgical success, especially in osteoporotic patients. However, these promising results must be interpreted with caution given the limitations of our study, including the use of supraphysiological dosing, a non-osteoporotic animal model, and a relatively short follow-up period. The potential cardiovascular risks associated with romosozumab administration in humans also warrant careful consideration when evaluating its clinical applicability.

Future studies should address these limitations through dose-optimization studies, the use of osteoporotic models, and longer follow-up periods to better understand the long-term effects and safety profile. Comparative studies with established bone-enhancing treatments such as teriparatide would also provide valuable context for romosozumab's relative efficacy in spinal fusion applications. These findings underscore the therapeutic promise of romosozumab not only in spinal surgery but also as a broader intervention for bone repair and healing. While our mechanistic understanding of romosozumab's effects on bone healing remains incomplete, further research is needed to explore its dose-response relationship, long-term safety, and efficacy in osteoporotic models. Moreover, the use of biochemical markers and microstructural analyses will help elucidate the underlying mechanisms of its action. With its demonstrated ability to enhance both structural and functional bone properties, romosozumab offers a promising avenue for advancing spinal surgery and improving patient outcomes, though a balanced assessment of benefits and risks will be essential for its optimal clinical implementation.
